# Trasmembrane chemokines CX3CL1 and CXCL16 drive interplay between neurons, microglia and astrocytes to counteract pMCAO and excitotoxic neuronal death

**DOI:** 10.3389/fncel.2014.00193

**Published:** 2014-07-10

**Authors:** Maria Rosito, Clotilde Lauro, Giuseppina Chece, Alessandra Porzia, Lucia Monaco, Fabrizio Mainiero, Myriam Catalano, Cristina Limatola, Flavia Trettel

**Affiliations:** ^1^Department of Physiology and Pharmacology, Istituto Pasteur Fondazione Cenci Bolognetti, Sapienza University of RomeRome, Italy; ^2^Department of Experimental Medicine, Sapienza University of RomeRome, Italy; ^3^IRCSS NeuroMedPozzilli, Italy

**Keywords:** CX3CL1, CXCL16, CCL2, A3R, glia cross-talk, neuroprotection, ischemia, excitotoxicity

## Abstract

Upon noxious insults, cells of the brain parenchyma activate endogenous self-protective mechanisms to counteract brain damage. Interplay between microglia and astrocytes can be determinant to build a physiological response to noxious stimuli arisen from injury or stress, thus understanding the cross talk between microglia and astrocytes would be helpful to elucidate the role of glial cells in endogenous protective mechanisms and might contribute to the development of new strategy to mobilize such program and reduce brain cell death. Here we demonstrate that chemokines CX3CL1 and CXCL16 are molecular players that synergistically drive cross-talk between neurons, microglia and astrocytes to promote physiological neuroprotective mechanisms that counteract neuronal cell death due to ischemic and excitotoxic insults. In an *in vivo* model of permanent middle cerebral artery occlusion (pMCAO) we found that exogenous administration of soluble CXCL16 reduces ischemic volume and that, upon pMCAO, endogenous CXCL16 signaling restrains brain damage, being ischemic volume reduced in mice that lack CXCL16 receptor. We demonstrated that CX3CL1, acting on microglia, elicits CXCL16 release from glia and this is important to induce neroprotection since lack of CXCL16 signaling impairs CX3CL1 neuroprotection against both *in vitro* Glu-excitotoxic insult and pMCAO. Moreover the activity of adenosine receptor A3R and the astrocytic release of CCL2 play also a role in trasmembrane chemokine neuroprotective effect, since their inactivation reduces CX3CL1- and CXCL16 induced neuroprotection.

## Introduction

Glial cells, able to sense changes in brain environment, represent active players in various pathological conditions such as chronic neurodegenerative disease, trauma and stroke. It is now established that both microglia and astrocytes can play dual roles in the CNS having either detrimental or beneficial effects participating and enhancing inflammatory conditions, or limiting neuroinflammation, favoring repair and enhancing neuronal survival (Liu et al., 2011). Thus understanding the cross talk between microglia and astrocytes would be helpful to elucidate the role of glial cells in pathological conditions.

Microglia-astrocytes interplay is granted by different types of soluble mediators including ATP, adenosine, glutamate (Glu) (Boison et al., 2010; Burnstock et al., 2011; Franke et al., 2012; Pascual et al., 2012), growth factors and inflammatory cytokines (Hamby and Sofroniew, 2010). We have recently shown that the transmembrane chemokine CXCL16 and its receptor, CXCR6, are constitutively expressed in glia and neurons being able to drive neuroprotection against Glu excitotoxicity and oxygen glucose deprivation (OGD) insults in culture (Rosito et al., 2012). In particular we found that the neuroprotective activity of CXCL16 involves astrocytic release of CCL2 and the synergistic activity of adenosine and adenosine type 3 receptor (A3R) on astrocytes.

The other known trasmembrane chemokine CX3CL1 is constitutively expressed in the brain only by neurons, while its unique receptor CX3CR1 is exclusively present on microglial cells. Recently described as a neuronal “off signal” that keep microglia in resting state (Biber et al., 2007), in the last decade the role of CX3CL1-CX3CR1 signaling in modulating neuron viability has emerged in several studies on neurodegenerative and neuroinflammatory disease models (Soriano et al., 2002; Cardona et al., 2006; Huang et al., 2006; Dénes et al., 2008; Bhaskar et al., 2010; Fuhrmann et al., 2010; Lee et al., 2010; Cipriani et al., 2011). Moreover CX3CL1 ability to preserve neurons from excitotoxic insult has been shown both *in vitro* and *in vivo*: in particular CX3CL1 signaling in microglia determines the release of soluble factors, such as adenosine that, acting on the adenosine receptor type 1 (A1R), concur to neuroprotection against Glu excitotoxicity and cerebral ischemia (Limatola et al., 2005; Lauro et al., 2010; Cipriani et al., 2011; Catalano et al., 2013).

In the present paper we studied the interplay between trasmembrane chemokines and between glial cells in determining neuroprotection against excitotoxic insults. In particular we found that: (i) CXCL16 is able to reduce ischemic brain volume; (ii) following ischemic insults there is an overexpression of CXCL16; (iii) CXCL16 and CCL2 are released from glia upon CX3CL1 stimulation; and (iv) A3R and CXCR6 concur to CX3CL1 mediated neuroprotection.

## Materials and methods

### Animals

Procedures using laboratory animals were in accordance with the international guidelines on the ethical use of animals from the European Communities Council Directive of 24 November 1986 (86/609/EEC). C57BL/6J (*wt*) and homozygous cxcr6^*gfp/gfp*^ knock-in mice (Unutmaz et al., 2000) in which the coding region of the receptor has been substituted with the coding region of the Green Fluorescent Protein (GFP) were obtained from Jackson Laboratory (strain name B6.129P2-Cxcr6tm1Litt/J). A3R knockout mice (A3R^–/–^) (Salvatore et al., 2000) were also used. Animals of either sex were used.

### Permanent middle cerebral artery occlusion (pMCAO)

Male mice (25–28 g, 10–12 weeks) were anesthetized with intraperitoneal Equitensine at 3.5 ml/kg (39 mM pentobarbital, 256 mM chloral hydrate, 86 mM MgSO4, 10% ethanol v/v, and 39.6% propyleneglycol v/v). The right MCA was permanently occluded by electrocoagulation as described previously (Storini et al., 2006). Mice were maintained at 37°C during surgery and sacrificed 24 h after pMCAO.

### Intracerebroventricul (i.c.v.) injection

Recombinant mouse CXCL16 or mouse CX3CL1 (Peprotech) was dissolved in saline solution and intracerebroventricularly injected 30 min before pMCAO. For dose-response experiments, mice were injected with 15, 70 and 150 pmol CXCL16/2 μl. Anesthetized animals were immobilized on a stereotaxic apparatus (David Kopf Instruments) and injected in the right cerebral ventricle (1 mm lateral and 3 mm deep, according to the atlas of Paxinos and Franklin, 2004). A constant rate of infusion (0.2 μl/min) was maintained with a pump (KD Scientific).

### Brain ischemic volume measurement

The extent of ischemic area was evaluated 24 h after ischemia. Mice were deeply anesthetized with Equitensine and transcardially perfused with ice-cold PBS (20 ml), pH 7.4, and paraformaldehyde (PFA; 4%, 50 ml) in PBS. The brains were carefully removed from the skull and transferred in 4% PFA at 4°C overnight, then to PBS/30% sucrose at 4°C overnight, frozen in isopentane at −45°C for 3 min, and then stored at −80°C until use. Twenty μm coronal brain cryosections were cut serially at 320 μm intervals and stained with cresyl violet. Infarct volumes were calculated by integration of the infarct areas on each brain slice, as described previously (Storini et al., 2006).

### Primary hippocampal cultures

Primary hippocampal cultures were prepared from the brain of 0–2-day-old wild type (*wt*), cxcr6^*gfp/gfp*^ and A3R^–/–^ mice. In brief, after careful dissection from diencephalic structures, the meninges were removed and the hippocampi chopped and digested in 0.025% trypsin, in Hank’s balanced salt solution (HBSS) for 20 min at 37°C. Cells were mechanically dissociated and plated at a density of 2.5 × 10^5^ in poly-L-lysine coated plastic 24-well dishes, in serum-free Neurobasal medium supplemented with B27, 0.5 mM L-glutamine and 100 μg/ml gentamicin. Successively, cells were kept at 37°C in 5% CO_2_ for 10–11 days *in vitro* (DIV) with a twice a week medium replacement (1:1 ratio). With this method we obtained 60–70% neurons, 30–35% astrocytes, 4–5% microglia, as determined with β-tubulin III, glial fibrillary acidic protein (GFAP), and isolectin IB4 staining (Lauro et al., 2010).

### Oxygen glucose deprivation (OGD)

Primary hippocampal cultures (10–11 DIV) were exposed to OGD. Briefly, culture medium was replaced with modified Locke’s buffer (without glucose), bubbled with 95% N_2_/5% CO_2_, and transferred into an anaerobic chamber (Billups-Rothenberg MIC- 101) containing a mixture of 95% N_2_/5% CO_2_, and humidified at 37°C for 90 min. For the reperfusion conditions OGD was terminated by replacing the OGD medium with the original conditioned medium. For comparative purposes, control cultures were treated under normoxic conditions (95% O_2_/5% CO_2_) in complete Locke’s buffer supplemented with glucose (5.6 mM).

### Glu excitotoxicity

In primary hippocampal cultures (10–11 DIV) conditioned medium was removed and stored for later usage; neurons were washed and stimulated with Glu (100 μM, 30 min) in modified Locke’s buffer (without MgCl_2_ plus 1 μM glycine to stimulate all types of Glu receptors), in the presence or in the absence of recombinant mouse mCX3CL1 (100 nM, Peprotech). Under these experimental conditions, only neurons die (Chen et al., 2000; Rosito et al., 2012). After treatment, cells were re-incubated in the original conditioned medium for 18–20 h, treated with lysis buffer (0.5% ethylhexadecyldimethylammonium bromide, 0.28% acetic acid, 0.5% Triton X-100, 3 mM NaC1, 2 mM MgCl, in PBS pH 7.4) and counted in a hemocytometer for viability, as described (Volontè et al., 1994). Data were expressed as percentage of viable cells taking as 100% the number of viable cells in control cultures. Variability in the number of viable cells in control conditions never exceeded 10%.When necessary, cells were pre-treated with monoclonal mouse αCCL2 Ab (3 μg/ml, 30 min; R&D MAB479), rat IgG (3 μg/ml, 30 min; Santa Cruz Biotecnology sc-2032), 3-propyl-6-ethyl-5-[(ethylthio)carbonyl]-2phenyl-4-propyl-3-pyrinide carboxylate (MRS1523; 100 nM, Sigma) in culture medium; drugs were present also during and after Glu challenge.

### RNA extraction and analysis

Total RNA from ipsilateral (ischemic core and penumbra) and controlateral (corresponding areas) brain emispheres of pMCAO mice, from primary hippocampal mixed cell cultures (5 × 10^5^ cells), from primary astrocytes (2.5 × 10^5^) and microglial cells (2.5 × 10^5^), was extracted by the use of Trizol reagent (Invitrogen). Reverse transcription reaction was performed in a thermocycler (MJ Mini Personal Thermal Cycler; Biorad) using IScriptTM Reverse Transcription Supermix (Biorad) according to the manufacturer’s protocol. Real-time PCR (RT-PCR) was carried out in a I-Cycler IQ Multicolor RT-PCR Detection System (Biorad) using SsoFast EvaGreen Supermix (Biorad) according to the manufacturer’s instructions. The PCR protocol consisted of 40 cycles of denaturation at 95°C for 30 s and annealing/extension at 58°C for 30 s. For quantification analysis the comparative Threshold Cycle (Ct) method was used. The Ct values from each gene were normalized to the Ct value of β-actin or GAPDH in the same RNA samples. Relative quantification was performed using the 2−ΔΔCt method (Schmittgen and Livak, 2008) and expressed as fold change in arbitrary values. Primer sequences targeted against CXCL16 (BC019961.1, GenBank), mouse β-actin and GAPDH were as follows: CXCL16-forw. TCCTTTTCTTGTTGGCGCTG, CXCL16rev. CAGCGACACTGCCCCTGGT; β-actin-forw.AGAGGGAAATCGTGCGTGAC, β-actin-rev. CAATAGTGATGACCTGGCCGT; GAPDH- forw. TCGTCCCGTAGACAAAATGG, GAPDH-rev. CAAGGGGTTGAAGCTCAGAT.

### Glial primary cultures

Primary cortical glial cells were prepared from 0–2-day-old *wt* mice. Cerebral cortices were chopped and digested in 30 U/ml papain for 40 min at 37°C followed by gentle trituration. The dissociated cells were washed, suspended in DMEM with 10% fetal bovine serum (FBS; Gibco) and 2 mM L-glutamine and plated at a density of 9–10 × 10^5^ in 175 cm^2^ cell culture flasks. At confluence (10–14 DIV), glial cells were shaken for 2 h at 37°C to detach and collect microglial cells. Astrocytes which remained attached to the bottom of the flask were treated with trypsin and collected. These procedures gave almost pure (no more than 2% astrocyte contamination) microglial cell population, and astrocytes cell population (4–6% of microglia contamination), as verified by staining with GFAP and isolectin IB4.

### Microglia-Astrocyte co-cultures

After 10–14 DIV, 8 × 10^5^ microglial cells were re-plated and co-cultured for 48 h with astrocytes cells (8 × 10^5^) seeded on 24 mm transwell cell-culture inserts (pore size 0.4 μm; Corning Life Sciences) which allows traffic of small diffusible substances, but prevents cell contact. After co-cultures, cells were treated with vehicle or soluble CX3CL1 (100 nM) for 18 h and upon stimulation proteins from cells and conditioned medium was collected and analyzed for Western blot. For CCL2 ELISA and mRNA analysis 2.5 × 10^5^ astrocyte were re-plated on 12 mm transwell cell-culture inserts (pore size 0.4 μm; Corning Life Sciences) and co-cultured with 2.5 × 10^5^ microglial cells. After 48 h cells were treated with vehicle or soluble CX3CL1 (100 nM) for 18 h. For ELISA conditioned medium (c.m.) was collected and analyzed according to the manufacturer (R&D Systems), while for mRNA extraction microglia and astrocytes were collected from the different transwell compartments.

### Proteins preparation

C.m. from microglia-astrocytes co-cultures were collected and concentrated by ultrafiltering on Ym-10 membrane (Centricon; Millipore). For cell membrane proteins preparation, cells were washed with phosphate-buffered saline and lysed for 15 min on ice in hypotonic buffer containing 10 mM HEPES pH 8, 1.5 mM MgCl_2_, 1 mM DDT, 1 μg/ml leupeptin, 1 μg/ml aprotinin, and 1 mM phenylmethylsulfonyl fluoride. After centrifugation at 1500 rpm for 5 min at 4°C, supernatant were ultra-centrifuged at 55000 rpm for 60 min, 4°C, and pellet were suspended in NaCl 10 mM.

### Western blot analysis

Protein samples were separated on 10% SDS-polyacrylamide gel and analyzed by Western immunoblot using a mouse CXCL16 antibody (0.2 μg/ml; R&D System, AF503) and HRP-tagged rabbit anti goat IgG secondary antibody (1:2000; Dako), and subsequently detected using a commercial chemiluminescent assay (Immun-Star WesternC Kit; Bio-Rad). Densitometric analysis was performed with Quantity One software (Biorad). Interpretation of western-blot bands for CXCL16 was according to Gough et al. (2004).

### Statistical analysis

The data are expressed as the means ± SEM. Where appropriate *t*-test, or analysis of variance (ANOVA) was used: we performed the parametric one-way ANOVA or two-way ANOVA followed by specific multiple comparison, as described in detail in figure legends. A value of *p* < 0.05 was considered significant. All statistical analysis was done using SigmaPlot 11.0 Software.

## Results

### Soluble CXCL16 reduces the ischemic volume in mouse brain after pMCAO

**Figure 1:**
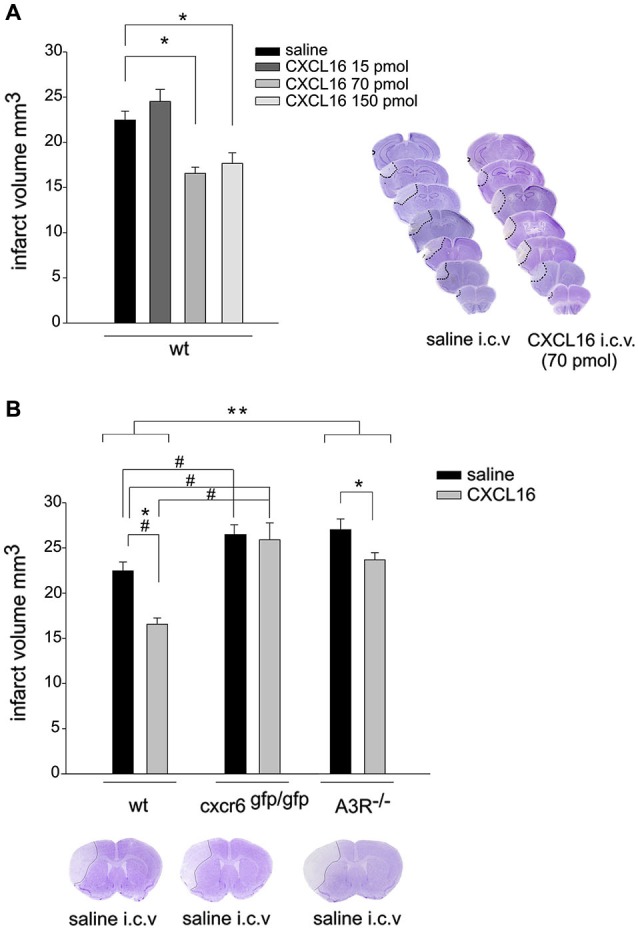
**CXCL16 reduces ischemic volume in pMCAO. (A)** CXCL16 dose-response effect. Left: Mice were i.c.v. injected with saline or CXCL16 (15–70-150 pmol) 30 min before pMCAO and analyzed for ischemic volume 24 h later (*n* = 4–10). Right: representative brain coronal sections from saline and CXCL16 i.c.v injected mice. Pale demarcated areas depict the ischemic lesions. (**B)** CXCL16 effect in *wt*, cxcr6 ^*gfp/gfp*^, and A3R^–/–^ mice. Mice of different genotypes (as indicated) were injected with saline or CXCL16 (70 pmol) before pMCAO and analyzed for ischemic volume (*n* = 6–10). Representative brain coronal section of pMCAO saline injected mice of different genotypes (Bottom). Results represent the mean ± SEM. Statistical analysis: one-way ANOVA followed by Holm-Sidak *post-hoc* test *p < 0.05 **(A)**. Two-way ANOVA followed by Holm-Sidak *post-hoc* test * *p* < 0.05; ** *p* < 0.001; * refers to *wt-*A3R^–/–^comparison; ^#^*p* < 0.05 refers to *wt*-cxcr6^*gfp/gfp*^ comparison **(B)**.

Since we have recently demonstrated that CXCL16 is neuroprotective against Glu excitotoxicity *in vitro* (Rosito et al., 2012), we now investigated the ability of CXCL16 to induce neuroprotection in mice upon pMCAO. In *wt* mice, i.c.v. injection of soluble CXCL16, 30 min before induction of pMCAO, resulted in a decreased ischemic volume compared to *wt* mice injected with saline (*n* = 10) control solution: in particular, a significant reduction in ischemic volume was observed after injection of 70 and 150 pmol of CXCL16 (*n* = 4–8; *p* < 0.05), while no effect was observed upon injection of 15 pmol (*n* = 4; Figure 1A). Further experiments were performed at 70 pmol. The neuroprotective effect of CXCL16 was specific, being absent in mice that lack CXCR6 receptor (cxcr6^*gfp/gfp*^ mice; Figure 1B). Two-way ANOVA analysis indicated a significant interaction between genotypes and treatments (*p* = 0.02) and *post hoc* evaluation revealed that CXCL16 was ineffective in reducing ischemic volume in cxcr6^*gfp/gfp*^ mice (*n* = 6). In addition, in cxcr6^*gfp/gfp*^ mice, pMCAO induced a significantly increased ischemic volume compared to *wt* animals suggesting that endogenous CXCL16-CXCR6 signaling contributes to restrain brain damage following ischemic insult.

To investigate whether the protective effect of CXCL16 upon pMCAO requires the activity of A3R, we analyzed the effect of i.c.v. administration of CXCL16 in A3R^–/–^ mice (*n* = 6–7; Figure 1B). Two-way ANOVA analysis reveals a significant differences between genotypes (*p* < 0.001), being the ischemic volume higher in A3R^ −/–^ vs. *wt* animals. CXCL16 administration was effective in reducing ischemic volume in both genotypes, (*p* < 0.05) but the reduction observed in A3R^–/–^ mice was less pronounced (12.3% in A3R^–/–^ vs. 26.3 % in *wt*).

### Ischemic insults induce up-regulation of endogenous CXCL16

**Figure 2:**
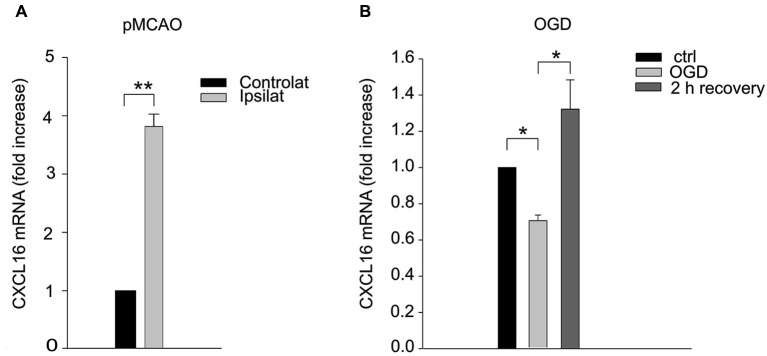
**CXCL16 expression is increased upon ischemic insults. (A)** CXCL16 mRNA quantification in pMCAO brain. qRT-PCR analysis in contralateral and ipsilateral emisphere 24 h after pMCAO (*n* = 5). **(B)** CXCL16 mRNA quantification in *in vitro* OGD. qRT-PCR analysis in primary hippocampal cultures after 90 min of OGD (*n* = 11) and after 2 h of recovery (*n* = 8). Data are expressed as mRNA fold increase normalized to β-actin. Results represent the mean ± SEM. Statistical analysis: Student’s *t*-test ** *p* < 0.001 **(A)**; one-way ANOVA followed by Dunn’s *post-hoc* test * *p* < 0.05 **(B)**.

Since CXCL16 signaling is determinant in reducing brain damage, we measured CXCL16 expression in the brain upon ischemia. RT-PCR analysis revealed that 24 h after pMCAO CXCL16 mRNA specifically increased in the ipsilateral hemisphere (*n* = 5; *p* < 0.001; Figure 2A), while no differences were observed in sham operated mice (not shown). Similar results were obtained *in vitro*, when hippocampal cultures were treated to induce OGD cell death (Rosito et al., 2012). After 90 min of OGD we observed a reduction of CXCL16 mRNA, followed by a significant increase after 2 h of recovery (*n* = 8–11; *p* < 0.05; Figure 2B).

### CXCL16 is released from microglia and astrocytes upon CX3CL1 stimulation

**Figure 3:**
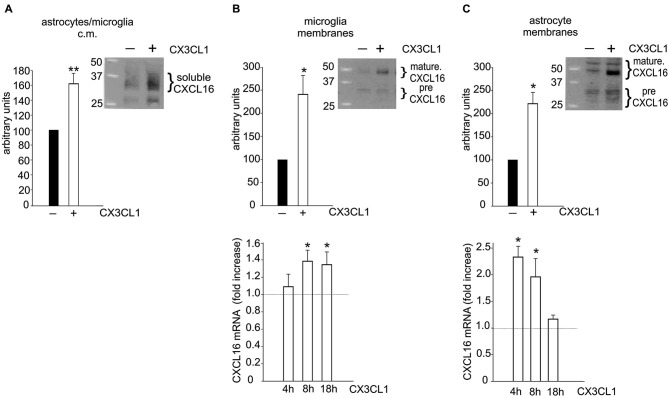
**Glial cells increase CXCL16 expression upon CX3CL1 stimulation. (A)** CX3CL1 induces the release of soluble CXCL16 from glial cells. Western-blot analysis for CXCL16 protein in the conditioned medium (c.m.) of astrocytes-microglia co-cultures stimulated or not with CX3CL1 (100 nM, 18 h). Representative blot is shown (*n* = 12). **(B–C)** CXCL16 up-regulation in microglia **(B)** and astrocytes **(C)** following CX3CL1 stimulation. Top: Western-blot analysis for mature and precursor (pre) CXCL16 species (Gough et al., 2004) in unstimulated and stimulated cells derived from co-cultures experiments. Representative blots are shown (*n* = 6). Bottom: qRT-PCR analysis for CXCL16 mRNA expression in microglia **(B)** and astrocytes **(C)** derived from co-cultures experiments treated or not with CX3CL1 for 4, 8, 18 h (*n* = 8). Data are expressed as mRNA fold increase normalized to GAPDH. Results represent the mean ± SEM. Statistical analysis: Student *t*-test * *p* < 0.05; ** *p* < 0.001 **(A, B–C** top panels); one-way ANOVA followed by Dunn’s *post hoc* test **(B–C)** bottom panels * *p* < 0.05.

To investigate if CXCL16 could be released from glia upon treatment with the neuroprotective chemokine CX3CL1, conditioned media (c.m.) from microglia/astrocytes co-cultures (in transwell system, see Section Materials and Methods) treated or not with CX3CL1 (100 nM, 18 h), were analyzed for CXCL16 presence. Data shown in Figure 3A revealed a significant increase in soluble CXCL16 upon CX3CL1 treatment (*n* = 12; *p* < 0.001). Membrane fractions of both microglia and astrocytes were also analyzed and we found that after CX3CL1 treatment the mature form of CXCL16 was significantly increased (Figures 3B,C top panels; *n* = 6; *p* < 0.05). Interestingly, we also observed an increased expression of CXCL16 mRNA upon CX3CL1 stimulation both in microglia and astrocytes (Figures 3B,C bottom panels; *n*= 6–8; *p* < 0.05).

### CXCL16 is a mediator of CX3CL1-induced neuroprotection against Glu excitotoxicity

**Figure 4:**
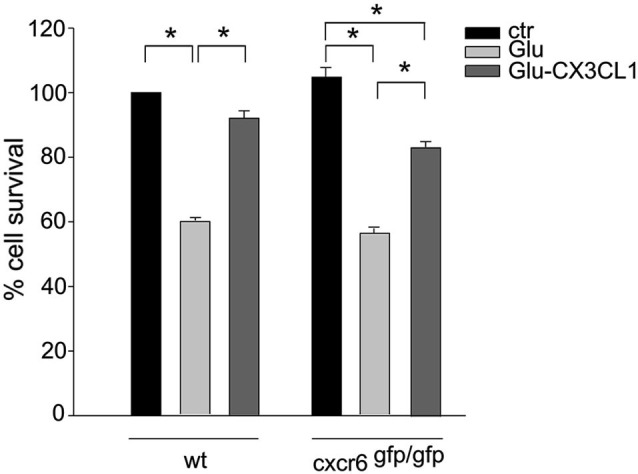
**CXCL16 contributes to CX3CL1 neuroprotection against Glu toxic insult.** Glu-excitotoxic experiments were performed in *wt* and cxcr6 ^*gfp/gfp*^ mice hippocampal cultures treated or not with CX3CL1 (*n* = 8–10). Data are expressed as percentage of viable cells in treated cultures taking as 100% the number of viable cells in *wt* control condition. Results represent the mean ± SEM. Statistical analysis: two-way ANOVA followed by Dunn’n *post-hoc* test * *p* < 0.05.

Since CXCL16 acts on its unique receptor CXCR6, we performed experiments on the neuroprotective activity of CX3CL1 against Glu excitotoxicity in hippocampal cultures obtained from cxcr6^*gfp/gfp*^ mice, to investigate the possible involvement of CXCL16 in CX3CL1-induced neuroprotection. As reported in Figure 4, CX3CL1 was less effective in preventing cell death in hippocampal cultures derived from mice that lack CXCL16 signaling, compared with *wt* cultures. In particular, statistical analysis (two-way ANOVA) indicated a significant interaction between genotypes and treatment, with a main effect of treatments (*p* = 0.004). In cxcr6^*gfp/gfp*^ mice, a significant difference between Glu and both control and Glu/CX3CL1 treated cells was observed (*n* = 8–10; *p* < 0.05).

### The mediators of CXCL16 neuroprotective activity are active players in CX3CL1 neuroprotection

**Figure 5:**
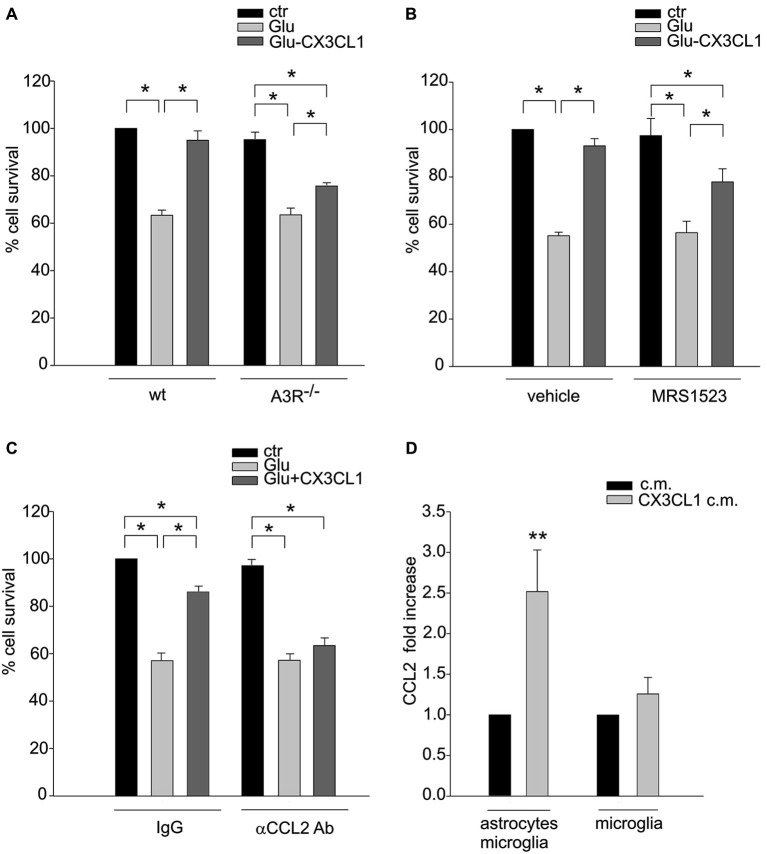
**A3R activity and astrocytic CCL2 concur to CX3CL1 neuroprotection. (A)** Genetic deletion of A3R reduces CX3CL1 neuroprotection. Glu-excitotoxic experiments were performed in hippocampal cells derived from *wt* or A3R^–/–^ mice (*n* = 6). Data are expressed as percentage of viable cells in treated cultures taking as 100% the number of viable cells in *wt* control condition. **(B)** Pharmacological inhibition of A3R reduces CX3CL1 neuroprotection. Primary hippocampal cells were treated with A3R specific antagonists MRS1523 and used for Glu-excitotoxic experiments (*n* = 5). Data are expressed as percentage of viable cells in treated cultures taking as 100% the number of viable cells in vehicle control condition. **(C)** Neutralization of CCL2 activity prevents CX3CL1 neuroprotection. Glu-excitotoxic experiment were performed in hippocampal cultures in the presence of neutralizing αCCL2 Ab (3 μg/ml) or control IgG (3μg/ml) (*n* = 4–6). Data are expressed as percentage of viable cells in treated cultures taking as 100% the number of viable cells in IgG control condition. **(D)** CX3CL1 triggers CCL2 release from astrocytes. Microglia-astrocytes co-cultures or microglia alone were treated with CX3CL1or vehicle, and the c.m. were collected after 18 h. CCL2 levels in the media were measured by ELISA (*n* = 7–11). Results represent the mean ± SEM. Statistical analysis: two-way ANOVA followed by Holm-Sidak *post-hoc* test * *p* < 0.05 **(A)**; one-way ANOVA followed by Holm-Sidak *post-hoc* test * *p* < 0.05 **(B–C)**; Student’s *t*-test ** *p* < 0.001 **(D)**.

The activity of CXCL16 and A3R on astrocytes and the consequential release of CCL2 are key events in CXCL16 induced neuroprotection (Rosito et al., 2012). To further corroborate the CX3CL1-CXCL16 connection in neuroprotection, we analyzed the contribution of A3R and CCL2 in this mechanism. We performed excitotoxic experiments in hippocampal cultures derived from A3R^–/–^ mice: at difference with *wt*, in A3R^–/–^ cultures CX3CL1 was less effective in preventing cell death (Figure 5A). Statistical analysis (two-way ANOVA) indicated a significant interaction between genotypes and treatment (*p* = 0.025), with a main effect of treatments. Both in *wt* and A3R^–/–^ animals, a significant difference between Glu and both control and Glu/CX3CL1 treated cells was observed (*post hoc* analysis, *n* = 6; *p* < 0.05). According to this result, the A3R inhibitor MRS1523 reduced CX3CL1 neuroprotection (*n* = 5; *p* < 0.05; Figure 5B). Moreover, as reported in Figure 5C in the presence of neutralizing αCCL2 Ab (but not with control IgG, both used at 3 μg/ml), CX3CL1 was not able to induce neuroprotection (*n* = 4; *p* < 0.05).

To verify the hypothesis that CX3CL1 could also induce the release of CCL2, that concur to neuroprotection, we stimulated microglia-astrocytes co-cultures or microglia with CX3CL1 for 18 h and measured CCL2 level in the c.m. Figure 5D shows a basal release of CCL2 that increases upon CX3CL1 stimulation in microglia-astrocyte co-culture (*n* = 11; *p* < 0.001 Rank sum Test) but not in microglia alone (*n* = 7; *p* = 0.7 Rank sum Test), suggesting that CX3CL1 acting on microglia, induces the release of CCL2 from astrocytes.

### CX3CL1 neuroprotection against pMCAO is reduced in cxcr6^*gfp/gfp*^ mice

**Figure 6:**
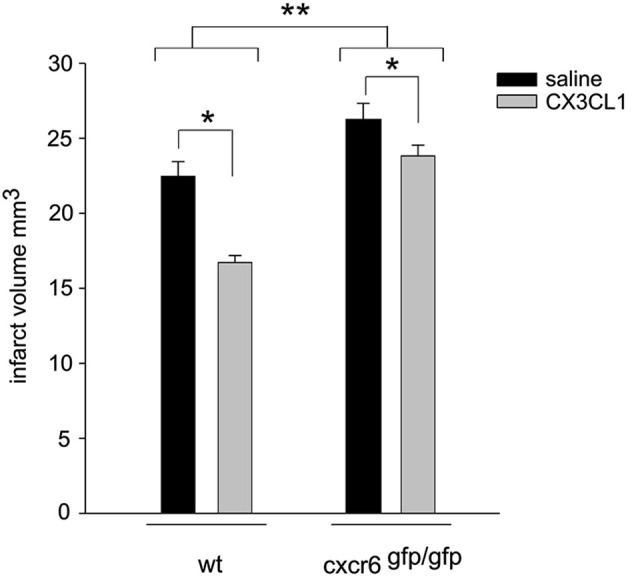
**CX3CL1 neuroprotection against pMCAO is reduced in cxcr6 ^*gfp/gfp*^ mice**. Mice were i.c.v. injected with saline or CX3CL1 (70 pmol) 30 min before pMCAO and analyzed for ischemic volume 24 h later (*n* = 4–10). Results represent the mean ± SEM. Statistical analysis: two-way ANOVA followed by Holm-Sidak *post-hoc* test * *p* < 0.05; ** *p* < 0.001.

CX3CL1 is neuroprotective in pMCAO (Cipriani et al., 2011). To further confirm that CXCL16 contributes to CX3CL1 neuroprotection, *wt* and cxcr6^*gfp/gfp*^ mice were i.c.v. injected with soluble CX3CL1, 30 min before induction of pMCAO: as reported in Figure 6, two-way ANOVA analysis reveals a significant difference between genotypes (*p* < 0.001), being the ischemic volume higher in cxcr6^*gfp/gfp*^ mice vs. *wt* animals. CX3CL1 administration was effective in reducing ischemic volume in both genotypes (*p* < 0.05) but the reduction observed in cxcr6^*gfp/gfp*^ mice was less pronounced being 10.9% (*n* = 6) vs. 25.6 % in *wt* (*n* = 4).

## Discussion

Glial cells, long thought to act as a mere “support” network, have been gaining increasing attention as crucial protagonists in a variety of neural functions including information processing but also cell viability. In the present paper we describe for the first time the ability of trasmembrane chemokines CX3CL1 and CXCL16 to drive molecular interplay between neurons, microglia and astrocytes in determining the neuroprotection against pMCAO and excitotoxic damage, showing that a concerted action of these cells is important to determine neuronal survival upon exposure to high level of Glu, a condition that normally occurs following ischemia (Castillo et al., 1996) but also in traumatic brain injuries (Zauner et al., 1996) or chronic neurodegenerative diseases (Shaw et al., 1995; Hallett and Standaert, 2004; Lipton, 2005).

**Figure 7:**
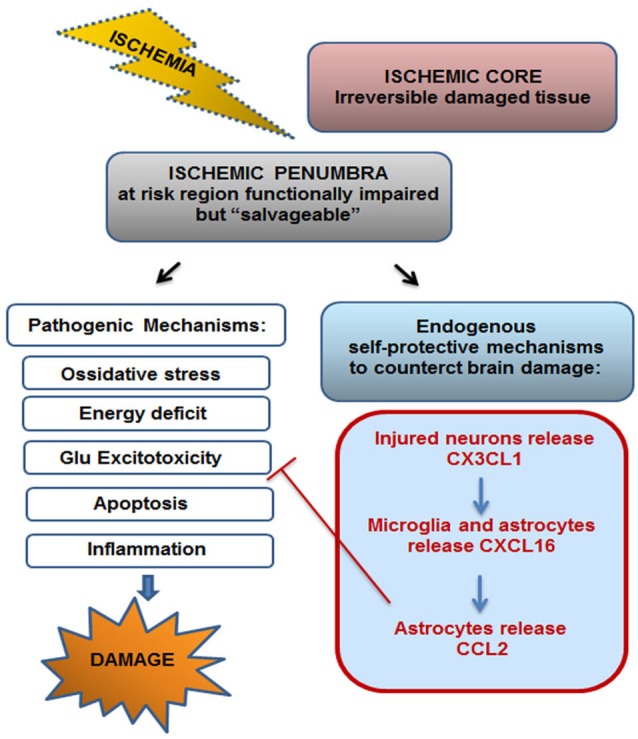
**Endogenous self-protective mechanism drived by CX3CL1–CXCL16-CCL2 upon ischemia**. Ischemic insult drives irreversible brain damage in ischemic core, while in the surrounding region (ischemic penumbra) brain tissue results to be functionally compromised but potentially “salvageable”. Upon ischemic insult both pathogenic and self-protective mechanisms occur in the penumbra. CX3CL1-CXCL16-CCL2 cascade, triggered by neurons-microglia-astrocytes cross-talk, represents one endogenous self-protective mechanism that contributes to the restriction of brain damage by counteracting Glu induced excitotoxicity.

In line with previous *in vitro* findings, we demonstrated that exogenous administration of soluble CXCL16 reduced brain ischemic volume following pMCAO; moreover we found that upon ischemic insult CXCL16 expression is increased in the ischemic hemisphere and that endogenous CXCL16 signaling is important *per se* to counteract brain damage, since in cxcr6^*gfp/gfp*^ mice there is a significant increase in brain ischemic volume upon pMCAO. All together, these data indicate that CXCL16 represents a physiological mediator of self-protective mechanisms engaged by brain parenchyma to restrain cell damage following toxic insult. Upon brain ischemia, there is the simultaneous activation of destructive pathways leading to cell death but also of local protective mechanisms. Although the damaging effectors apparently prevail, evidences suggest that concomitant self-protective mechanisms might limit the resulting damage and set the stage for tissue repair and reorganization (Moskowitz et al., 2010; Iadecola and Anrather, 2011; Figure 7). Thus unveiling the molecular players that act in self-protective mechanism might provide new opportunity to treat brain pathologies.

Damaged neurons respond to neurotoxic insults releasing soluble factors that can be sensed by surrounding glia: CX3CL1, a chemokine selectively expressed by neurons in the nervous system, is one of such mediators being upregulated (Tarozzo et al., 2002; Zhu et al., 2009), cleaved and released upon ischemia and excitotoxic insult (Chapman et al., 2000; Limatola et al., 2005; Noda et al., 2011 ) and being able to drive neuroprotection (Limatola et al., 2005; Lauro et al., 2010; Cipriani et al., 2011). We reported here that, upon CX3CL1 stimulation, glial cells produce and release CXCL16, important for CX3CL1 neuroprotective effect. We speculate that CX3CL1 released from neurons upon ischemia might drive microglia-astrocytes cross-talk leading to CXCL16 increase. These data further corroborate the idea that, although the only direct targets of CX3CL1 are microglial cells, its neuroprotective effects are mediated by engagement of astrocytes that concur to limit excitotoxic cell death with the synergistic activity of adenosine (Catalano et al., 2013).

We do not know the mechanism that leads to the release of CXCL16 from glia, however it has been recently reported that activation of the purinergic receptor P2X7 induces CXCL16 shedding from RPMI8226 myeloma B cells (Pupovac et al., 2013). Hippocampal cells stimulation with CX3CL1 induces an increase in extracellular adenosine probably derived from released ATP, the effect being specifically blocked by the treatment with the ectonucleotidase inhibitor alpha-beta-methyleneadenosine 5′-diphosphate sodium salt (AOPCP) (Lauro et al., 2010). Since both astrocytes and microglia express P2X7 receptors, it could be hypothesized that CX3CL1 induces ATP release from microglia that, acting on P2X7 receptors, induces CXCL16 shedding from surrounding glial cells. A role for P2X7 in the release of neuroprotective mediators is in agreement with previous data showing that P2X7 activation reduces excitotoxic neuronal death, through TNF-α shedding from microglia (Suzuki et al., 2004).

Adenosine modulates neuron-glia communication (Boison et al., 2010) and can mediate neuroprotective effects through the activity of its own receptors: in this regards the activity of A1R is crucial to allow neuroprotection driven by CX3CL1, IL-6, oncostatin M (OSM), BDNF and erythropoietin (EPO) (Biber et al., 2008; Lauro et al., 2010; Moidunny et al., 2010). Also A3R activity can mediate neuroprotection since it has been shown that hypoxic conditions determine a wider neurodegeneration in A3R^–/–^ mice (Fedorova et al., 2003) and i.c.v. injection of A3R selective agonist in mice reduces brain ischemic volume (Chen et al., 2006). In the present work, we confirmed an increased ischemic volume in A3R^–/–^ mice compared to *wt* mice and found that the ability of CXCL16 to reduce ischemic volume is less pronounced in these mice. This is in line with our previous *in vitro* findings, where we have shown that soluble CXCL16 is able to promote neuronal survival against excitotoxic damage depending on A3R activity (Rosito et al., 2012), and in particular the synergistic activity of CXCL16 and A3R on astrocytes causes the release of CCL2 that act as a key mediator of neuroprotection.

We speculate that upon ischemic insult, CXCL16 released from glia concurs to endogenous neuroprotective mechanism elicited by neuronal CX3CL1 since we found that CX3CL1-induced neuroprotection was reduced in cxcr6^*gfp/gfp*^ mice; both genetic and pharmacological inactivation of A3R reduces CX3CL1 neuroprotection against Glu excitotoxic insult; CX3CL1 is able to increase the release of CCL2 from astrocytes; CCL2 activity is important for CX3CL1 protective effect.

Nevertheless, our data showed that impairment of CXCL16 or A3R signaling in transgenic animals reduced, but did not totally prevented CX3CL1 neuroprotection, indicating that the mechanism we here proposed represent only a portion of the neuroprotective mechanisms driven by CX3CL1.

The involvement of A1R in CX3CL1 neuroprotection (Lauro et al., 2010) strongly suggests that there must be at least another mechanism, independent from CXCL16, important to protect cells from Glu excitoxicity: accordingly we have recently published that the activity of Glu transporter GLT1 on astrocytes is increased by CX3CL1, with mechanisms requiring A1R activation and this event is also crucial for CX3CL1 neuroprotection (Catalano et al., 2013).

In conclusion, the present work highlights the role played by chemokines as key endogenous modulators of the cross-talk between cells of brain parenchyma, that drive physiological neuroprotective mechanisms. In particular we demonstrated the existence of chemokine induced chemokine release (CX3CL1-CXCL16-CCL2) mechanism that involves neurons, microglia and astrocytes and that represents an endogenous self-protective mechanism that upon brain ischemia can limit cell damage in the ischemic penumbra, by counteracting neuronal cell death due to Glu excitotoxicity (Figure 7).

## Conflict of interest statement

The authors declare that the research was conducted in the absence of any commercial or financial relationships that could be construed as a potential conflict of interest.
